# Salivary Alpha-Amylase as a Biomarker of Stress in Behavioral Medicine

**DOI:** 10.1007/s12529-019-09843-x

**Published:** 2020-01-03

**Authors:** Nida Ali, Urs M. Nater

**Affiliations:** Clinical Psychology, Department of Psychology, University of Vienna, Liebiggasse 5, 1010 Vienna, Austria

**Keywords:** Autonomic nervous system, Salivary alpha-amylase, Behavioral medicine, Stress, Biomarkers

## Abstract

In recent years, research in behavioral medicine has become increasingly focused on understanding how chronic and acute exposure to stress impacts health outcomes. During stress, the body’s physiological stress systems are activated. These systems closely interact with the immune system and are, thus, importantly implicated in the onset and maintenance of disease states. While much of the research in behavioral medicine that has investigated the effects of stress on disease has focused on the role of the hypothalamic-pituitary-adrenal axis and its downstream biomarker, cortisol, it is evident that the autonomic nervous system (ANS) also plays a crucial role in both the biological stress process and the manifestation and maintenance of stress-related symptoms. In recent years salivary alpha-amylase (sAA) has emerged as a valid and reliable marker of ANS activity in stress research and is therefore an important biomarker to consider in behavioral medicine. In this commentary, we will highlight research relevant for behavioral medicine that has utilized sAA measurements, both basally, and in response to stress, to examine ANS function in clinical populations. We will additionally summarize findings from studies that have examined the effects of various targeted interventions on changes in sAA levels. Through this, our aim is to present evidence that sAA can serve as a feasible biomarker of ANS (dys)function in health and disease. To this end, we will also highlight important methodological considerations for readers to keep in mind when including sAA assessments in their own studies. The overarching goal of this brief commentary is to highlight how a multidimensional approach toward physiological stress measurement can allow researchers to develop a better understanding of physical health and disease states.

## Introduction

Stress is a pervasive phenomenon that accompanies the everyday lives of humans and animals. Under acute conditions, the experience of stress is an important starting point from which resources are activated that enable an organism to tackle the presenting threats and challenges appropriately. However, the longer the experience of stress persists, the more likely it is that deleterious effects might occur, ultimately leading to negative (health) consequences. In this regard stress has been shown to precipitate, exacerbate, and perpetuate both psychological and physical illness [1, 2] and is thus a fundamental target of investigation in the behavioral medicine literature [3].

During stress, the body’s physiological and psychological responses are activated to help organisms adaptively cope with the stressful situation. At the physiological level, this encompasses activation and coordination among both the central and the peripheral nervous systems. The central nervous system response involves activation of the hypothalamus and the brainstem, while the peripheral components include outflow from the hypothalamic-pituitary-adrenal (HPA) axis and the autonomic nervous system (ANS) – which comprises the efferent sympathetic-adrenal-medullary (SAM) system and parasympathetic system. Together these systems interact with the immune system and are therefore closely involved in the pathogenesis of stress-related diseases [4]. In the context of stress research in behavioral medicine, the HPA axis is the most widely studied neuroendocrine stress system [5], and consequently, its downstream hormone, cortisol, has become the “gold standard” biomarker by which to evaluate systemic fluctuations of the HPA axis. Cortisol has additionally received increased attention because it can easily and reliably be measured from saliva. This has resulted in the greater inclusion of salivary cortisol as the method of choice in studies probing the effects of stress on various health outcomes. Thus, while the role of cortisol in stress and related pathological states has been widely studied, it is evident that the ANS also plays a crucial role in both the biological stress process and the manifestation and maintenance of stress-related symptoms. While there are a number of ANS markers that are indicative of autonomic activation, different markers have been found to reflect different aspects of ANS activity, and as such their utility is dependent on the specific mechanism under study (for a review see [6]). Heart rate variability, as an example, is a widely used laboratory assessment for cardiovascular functioning, since it reflects input of sympathetic and parasympathetic control of the heart [7]. However, technological and experimental complexities associated with the measurement of heart rate variability restrict its ability to assess ANS functioning across a wide range of settings and in different populations [8]. Likewise, other ANS markers such as centrally synthesized catecholamines, norepinephrine and epinephrine, require invasive blood or spinal fluid draws, which too might limit their utility, particularly in controlled research settings.

Over the past 15 years or so, salivary alpha-amylase (sAA) has emerged as a valid and reliable marker of ANS activity in stress research [9]. Alpha-amylase is a salivary enzyme that is involved in the digestion of carbohydrates and starches. It can be measured quickly and noninvasively through saliva collection and, as such, in the acute stress literature, is frequently used as a proxy measure of sympathetic arousal particularly in studies looking to reduce participant load or when paradigms require participants to collect samples at home. It is important to note that there is some debate in the literature about whether sAA levels measured during stress reflect purely sympathetic or parasympathetic activity or some combination of both. However, there is some evidence that sAA may reflect central noradrenergic activation, as indicated by Nater and Rohleder [9] and more recently by Warren et al. [10] (also see [11, 12]). Thus, depending on the research context (i.e., whether acute stress has been induced or a pharmacological challenge has been used), sAA can broadly be considered to represent ANS activity [13] and can therefore be used as a biomarker to assess ANS functioning within the context of behavioral medicine. In the next sections, we will highlight studies, pertinent for behavioral medicine, that have used sAA as a marker of stress.

## Awakening and Diurnal sAA Activity

Salivary alpha-amylase, like cortisol, has a distinct diurnal profile. However, in contrast to cortisol (which reaches peak values within a half hour of awakening), sAA levels drop sharply in the first 30 min of awakening and then steadily rise over the course of the day [14]. Diurnal measures are typically assessed via multiple daily saliva samples collected either in a single day or across multiple days. In the behavioral medicine literature, studies have utilized multiple daily samplings to assess the integrity of the ANS in groups of interest through examination of the amylase awakening response (AAR) and/or the total diurnal output [15]. In this context, ANS dysregulation typically manifests itself as a blunted AAR (i.e., less of a decline 30 min after awakening) and higher sAA output throughout the day. Both of these measures can therefore serve as indices of pathological dysregulations of the ANS, such as, for example, in conditions associated with chronic stress [9, 14]. Consequently, studies have sought to examine sAA profiles in different at-risk and clinical samples that have been exposed to chronic levels of stress. For example, a study conducted in sexual minorities found that, compared to heterosexual women, lesbian or bisexual women had significantly blunted sAA at awakening and elevated sAA levels diurnally [16]. Importantly, the authors found that this pattern was not present in gay or bisexual men, suggesting differential sensitivities of the ANS between men and women exposed to stigma and chronic stress associated with their sexual orientation. A similar sex-specific pattern of ANS dysregulation was observed in a study conducted in nurses, a group that is considered to be at high risk for burnout since individuals are chronically exposed to stressors associated with their working conditions. Here, the authors found that compared to male nurses, female nurses showed more pronounced increases in sAA over the course of the day [17], again suggesting differential sensitivities of the ANS between men and women exposed to chronic levels of occupational stress. Other studies have examined psychosocial factors that are associated with occupational stress and have found that both psychological distress and reduced professional efficacy, or sense of accomplishment, can have important effects on basal ANS functioning. Here, lower sAA levels at awakening, but higher values in the afternoon, and at bedtime, were observed in office workers reporting high levels of distress [18] and, during periods of high stress, in professional jockeys experiencing low levels of professional efficacy [19]. sAA has also been included as a biomarker in studies investigating the physiological toll exacted by chronic stress experienced as a result of familial caregiving. Here too, attenuated diurnal profiles of sAA were observed in caregiving family members of patients with brain cancer [20] and dementia [21].

Clinical studies have additionally sought to investigate the utility of sAA as a biomarker to distinguish patient groups from controls. In one study, Wolf et al. (2008) found that compared to healthy controls, children with asthma had lower sAA levels throughout the day, with no differences between groups on cortisol levels. Moreover, higher levels of self-reported chronic stress in the asthma group were associated with lower diurnal sAA [22]. Similarly, in another study, diurnal sAA patterns, but not cortisol, successfully differentiated asthma patients with comorbid generalized anxiety – who had significantly higher diurnal sAA levels – from asthma patients without anxiety and healthy controls [23]. Finally, a study investigating diurnal changes in sAA at various stages of pregnancy found that women with a history of previous miscarriage had significantly flatter sAA trajectories throughout the day, compared to pregnant women with no previous miscarriages [24]. The overall results from these studies suggest that awakening and diurnal sAA can be potential biomarkers to investigate ANS dysregulations that are typically observed in chronic stress-related pathologies.

## sAA Reactivity to Stress

Another way in which the integrity of the ANS can be assessed is by putting the system through a “stress test.” This typically involves exposing participants to an in-lab psychosocial stress paradigm in order to investigate the consequential effects of the stress induction on biomarker reactivity. Previous work by Nater et al. (2005) in this domain has demonstrated the efficacy of using sAA to examine ANS responses to acute stress [25]. Within the context of behavioral medicine, only a few studies thus far have included sAA assessments to assess stress reactivity in at-risk or clinical samples. In one study, Fischer et al. (2017) investigated the effects of acute stress induced in the context of ethnic discrimination in a group of Turkish immigrants. Here the authors found that face-to-face exposure to derogatory remarks of a “physician” who expressed negative stereotypes about the participants’ ethnic group resulted in increased sAA reactivity, suggesting that ethnic discrimination has acute psychobiological effects in minority groups [26]. With respect to clinical conditions associated with chronic stress, two studies have examined the effects of acute stress on sAA reactivity. Here, compared to healthy controls, blunted sAA reactivity was observed in individuals diagnosed with burnout [27], as well in individuals with tinnitus (who did not differ from the controls in cortisol reactivity) [28]. Finally, a similar effect on sAA, but not cortisol, was observed in response to acute wound care procedures following burn injuries in a pediatric population [29]. Taken together, these findings demonstrate the differential effects of chronic stress on the sensitivity of the ANS (but not the HPA axis) in certain clinical states, further highlighting the importance of including sAA assessments in behavioral medicine research.

## sAA in Behavioral Intervention Studies

Studies have also utilized sAA assessments to determine the effectiveness of various interventions designed to alter the functioning of the stress system. To this end, in healthy samples, mind-body interventions such as stress reduction programs and self-compassion training have been shown to reduce sAA levels, diurnally albeit marginally [30], in reaction to stress [31], as well as in comparison to a pre-intervention measurement time-point [32]. Additionally, one study conducted in long-term meditators found that the quality of meditation practice was associated with differential sAA awakening responses, such that deeper meditation practices were associated with steeper (more negative) awakening slopes, compared to shallower practices [33]. Within patient populations, mindfulness-based interventions have also been shown to have dampening effects on sAA. One randomized trial investigated the effects of a 3-week sleep-focused mind-body intervention in cancer survivors. Here, the authors found that patients in the intervention group had significantly lower mean sAA awakening levels (but not cortisol), compared to their own baseline levels and to the active control condition [34]. Likewise, in a study conducted in functional somatic syndrome patients, a single autogenic relaxation training session resulted in significantly lower sAA in the patient group at the post-intervention assessment, compared to their own pre-intervention levels and to healthy controls [35]. Finally, de Brouwer et al. (2011) examined the effects of a brief (four sessions) stress management intervention on changes in stress reactivity, in patients with rheumatoid arthritis. Here, the authors found that at the 9-week post-intervention follow-up session, patients who were identified as being at high-risk for anxiety and depression showed marginally lower sAA reactive responses to stress, compared to high-risk individuals from the control group [36].

A few studies have examined the effects of other forms of stress reduction interventions, such as yoga, in pregnant women, given that pregnancy is associated with important changes in the ANS that can have significant effects on the health of mothers and infants [37]. In one such study, pregnant women attending prenatal yoga classes at gestational ages 20–23 weeks, 28–31 weeks, and 36–40 weeks, provided saliva samples before and after attending each class. The authors found that sAA levels after each yoga session were significantly lower than the sAA values obtained at the beginning of the class and that this effect was consistent across all gestational ages [38]. Another study similarly found that mean sAA values decreased significantly in pregnant women after each of the two yoga classes attended, at 27–32 as well as at 34–37 gestational weeks [39].

Finally, studies investigating the effects of other forms of interventions have found similar reductions in sAA levels in response to the treatments. For example, in one study, a 4-week “warm touch”-based support intervention delivered to married couples was associated with significantly lower diurnal sAA (but not cortisol), compared to the couples’ pre-intervention baseline levels [40]. Likewise, Inagaki and Eisenberger (2016) found that compared to a control condition, individuals who were randomly assigned to a “support giving” condition, which encompassed writing an encouraging note to a friend, showed reduced sAA reactivity in response to psychosocial stress. Importantly, here too the support-giving intervention was associated with lower sAA reactivity to stress, but no effects were observed for cortisol [41]. Finally, a study conducted in a sample of inpatient infants found that distraction from negative emotions in the form of nurses’ uniforms (animal patterns and geometric shapes versus plain white) was associated with significantly lower sAA reactivity in response to a mildly stressful medical procedure [42]. The overall results from these studies demonstrate that the administration of stress-reduction interventions typically result in lower (basal and stress reactive) sAA levels, often with no effects on cortisol, thus highlighting the importance of including sAA assessments in studies aiming to investigate the efficacy of health improvement programs on the functioning of the stress system.

## Conclusion

The studies described in the previous sections highlight the efficacy of including sAA as a biomarker in behavioral medicine. The findings demonstrate that sAA secretion patterns such as blunted levels at awakening, and hyper- or hypo-secretions diurnally, and/or in reaction to acute stress can serve as valuable indicators of the ANS dysregulations that typically occur in chronic stress related pathologies. In addition, sAA appears to be uniquely sensitive ( compared to cortisol for example) to interventions that aim to promote better health outcomes, in both at-risk and clinical samples. Thus, the integration of sAA within the context of stress assessment and intervention can advance our understanding of how the stress systems interact and how they can impact behavioral and health outcomes in clinically relevant populations.

However, despite its growing popularity in stress research, the number of studies in behavioral medicine that include sAA assessments remains fairly small. To this end, one aim of this commentary has been to present evidence that sAA can be a feasible biomarker of ANS (dys)function in health and disease. While our goal here was to simply present the available evidence, a more comprehensive and systematic review is necessary to critically evaluate the quality of the studies presented and highlight their strengths and limitations (such as for example, small sample sizes, and variance in the strengths of the effects reported in these studies).

As an additional goal, we hope to have convinced researchers of the importance of including sAA assessments in future studies, given the crucial role of the ANS in numerous disease states. This is especially pertinent in cases when researchers are already interested in or are collecting salivary cortisol measures in their studies. Since both cortisol and sAA can be analyzed from the same saliva sample, this additional analysis will allow researchers to measure activities of *both* HPA and ANS concomitantly [15]. Furthermore, the inclusion of sAA assessments can complement other psychophysiological measures, such as heart rate variability, and allow researchers to examine different components of the autonomic response in conjunction [43]. However, the added utility of sAA assessments should be considered based on the specific research questions being investigated; or if, for example, study protocols already include other ANS measures, such as HRV, skin conductance, or blood sampling for catecholamines; or if the inclusion of sAA will add significant participant and/or financial burden to the study.

Researchers interested in including sAA assessments in their studies should keep the following considerations in mind. First, sAA levels are sensitive to sampling techniques, specifically the type of oral fluid collected, which can vary based on whether stimulated or unstimulated saliva samples are provided. This is because different salivary glands contribute different rates of saliva secretion, which can influence the amounts of sAA secreted into oral fluids. Therefore, sAA results may vary depending on where in the mouth samples are collected and whether participants provide stimulated (e.g., chewing on cotton rolls) or unstimulated (e.g., via passive drool technique) saliva samples [44, 45]. Recent developments in sAA biosensor technology, which consists of disposable saliva collector strips and a hand-held reader, aim to address some of these methodological concerns and could be a potential option to consider, especially within clinical settings [46]. Secondly, with respect to participant characteristics, there is some evidence of developmental changes in sAA across early childhood, with studies showing that sAA levels are very low in newborn infants and continue to increase across late infancy and childhood. Acute stress studies have additionally shown that stress induction tasks do not elicit increases in sAA levels in newborns [47]. For a comprehensive list of methodological issues that should be considered in sAA assessments, the interested reader may also consult the recently published recommendations by Strahler et al. [15].

These considerations notwithstanding future studies should adopt a multimodal approach of assessment when examining the effects of stress on health. This is important given that the physiological stress system is a complex, multifaceted system that encompasses dynamic interplay between the ANS, HPA axis, and the immune system. Studies should thus consider including biomarkers of these different systems and include multiple sampling time-points within the same study, in order to better understand the interactions among the different systems and investigate how dysregulated cross talk among them can contribute to disease [48, 49]. In this regard, an emerging line of research has been investigating the molecular mechanisms that are involved in stress sensitivity and reactivity. Specifically, studies have examined the role of genetic influences and how they contribute to individual differences in sAA and potentially impact behavioral and health outcomes. For example, studies have found that individual differences in copy number variants for the AMY1 gene for sAA have been associated with differences in eating behaviors and taste preferences [50], body mass index [51], and risk for developing metabolic diseases [52]. This approach thus offers a promising avenue to further our understanding of the underlying molecular mechanisms that contribute to individual differences in the development, and maintenance, of disease states. Overall, our aim through this work has been to highlight how a multidimensional approach can better allow researchers to identify differential patterns that underlie individual differences in risk and resilience, in order to ultimately develop more sensitive diagnostic tools and targeted interventions in behavioral medicine.
